# Effect of sample storage on pH-value in equine faeces

**DOI:** 10.1093/tas/txag131

**Published:** 2026-08-25

**Authors:** Katrin Madelene Lindroth, Cecilia Elisabeth Müller

**Affiliations:** Department of Applied Animal Science and Welfare, Swedish University of Agricultural Sciences, Uppsala, 75007, Sweden; Department of Applied Animal Science and Welfare, Swedish University of Agricultural Sciences, Uppsala, 75007, Sweden

**Keywords:** acidity, faecal, hindgut, horse, stool

## Abstract

Measurements of pH-value in equine faeces are often performed in studies on nutrition and health in horses, but a standard protocol for it is lacking despite the knowledge that different sample handling may affect the pH-value. Sample handling prior to pH-measurement is often determined by practical settings and has been reported to vary in published studies within the area. The purpose of the current study was therefore to investigate the influence of different combinations of storage time and temperature on the pH-value in equine faecal samples in comparison to immediate pH-measurement at defecation. Faecal samples from 105 horses were collected, and each sample was divided into 14 sub-samples. The sub-samples were stored in room temperature (+20 to + 21°C), in a refrigerator (+4 to + 6°C) or in a freezer (-20°C) for different periods of time, after which pH was measured. Measurement of pH was performed immediately at defecation (reference value, RT0), after 2 h in room temperature (RT2), after 2, 4, 8, 12, 24 and 48 h in a refrigerator (RE2-RE48;), after 24, 48 and 72 h in a freezer (F24-F72), and after 1, 3 and 6 months in the same freezer (F1M-F6M). Measurements of pH were done on undiluted liquid pressed from whole faecal samples using a two-point calibrated pH-meter and was repeated three times on each sub-sample. Data were analysed using an ANOVA mixed model procedure with sample treatment (temperature and time) as a fixed effect and horse as a random effect, and with Dunnetts adjustment of *P*-values as all sample treatments were compared to the reference value at RT0. Results showed that mean pH ± SD at RT0 was 6.60 ± 0.04 and that it differed (*P* < 0.01) from all sample treatments except RT2, RE8 and RE48 (*P* > 0.34). All frozen samples had higher (*P* < 0.01) mean pH-values compared to RT0, and ranged from 6.65 ± 0.38 at F48 to 6.75 ± 0.40 at F72. All other frozen samples had pH-values between 6.65 and 6.75. Refrigerated samples RE2 and RE4 had lower (*P* < 0.001) mean pH-values (6.52 ± 0.36 for both) and RE12 and RE24 samples had higher (*P* < 0.001) mean pH-values (6.67 ± 0.34 and 6.67 ± 0.38, respectively) compared to RT0. The results of this study therefore showed that if pH cannot be measured immediately at defecation, storage of equine faecal samples in room temperature for 2 h or in a refrigerator at + 4 to + 6°C for 8 h or 48 h is another option, as pH at these timepoints did not differ from RT0.

## Teaser

Sample **s**torage time and temperature affect accuracy of pH-measurements in equine faeces.

## Introduction

Equine faecal pH is often used as an indicator of the health status within the equine gastrointestinal tract in health and nutrition studies (Johnson et al. 1998; Nicol et al. 2002; Hussein et al. 2004; Milinovich et al. 2007; Williamson et al. 2007; Swyers et al. 2008; Næsset and Austbø 2010; van den Berg et al. 2013; Hydock et al. 2014; Johnson and Rossow 2019; Davies et al. 2021; Lindroth et al. 2022). However, the difference in faecal pH between horses with and without gastrointestinal disturbances varies largely in different studies; faecal pH has been reported to be 6.64 vs. 7.04 (*P* < 0.05) in horses with and without cereal-induced hindgut acidosis (Hussein et al. 2004), while feeding two compared to eight kg of concentrates per day to horses resulted in mean faecal pH-values of 6.49 and 6.26, respectively (*P* > 0.05) (Williamson et al. 2007). In another study (Swyers et al. 2008) faecal pH decreased (*P* < 0.001) from 6.61 to 6.57 following increased starch content of the diet fed to the horses, and in another study (Berg et al. 2005) faecal pH decreased (*P* < 0.01) from 6.48 to 6.38 with diet supplementation of fructo-oligosaccharides. The difference in pH-units between horses considered to have a hindgut disturbance and control horses considered to not have such a disturbance has thus been reported to vary from 0.04 to 0.4 (Hussein et al. 2004; Berg et al. 2005; Williamson et al. 2007; Swyers et al. 2008). This range is lower or close to the standard deviation (SD) in pH in equine faecal samples handled or treated with different methods (Hydock et al. 2014; Tischer et al. 2023), indicating that the noise from sample handling or treatment may mask or make it challenging to detect differences in pH that are important from a gastrointestinal health perspective. Measurement of faecal pH is an easy, non-invasive, quick, and inexpensive tool, but it is important to reduce variation in the measurements that originates from sample handling. There is considerable variation in sample handling of equine faeces in studies where pH has been measured (Table 1), and the lack of standardized protocols for sampling and sample handling of equine faeces for pH measurements is evident (Hydock et al. 2014). This is a tangible drawback as it has been reported that the pH-value of equine faeces can be influenced by several factors involved in sampling, such as sampling location (rectal or post-defecation; Næsset and Austbø 2010), sample treatment such as dilution (Hydock et al. 2014), and the time period and sample storage conditions between sampling and pH-measurement (Tischer et al. 2023). In both clinical practice and research settings, there can be a delay between sampling of faeces and pH measurement for practical reasons, such as transport of samples to a laboratory, and samples may need to be stored for longer times prior to pH-measurements. Microbial production of short chain fatty acids (SCFA) as well as other biochemical changes may continue in the faecal sample after sampling which can influence the pH-value (Lowman et al. 1999). It is therefore important to understand how sample handling and sample storage conditions affect the pH-value in this matrix, and which sample storage conditions will have the least impact on pH-value. The aim of this study was therefore to systematically investigate how different storage times and temperatures influenced pH-value in equine faecal samples. The hypothesis was that storage of samples in room temperature, in a refrigerator and in a freezer would affect the pH-value compared to when pH was measured in the sample immediately at defecation.

**Table 1:** A selection of studies (in chronological order) where pH has been measured in equine faecal samples with variations in sampling and measurement protocols.

Reference	Sample treatment Ratio water: faeces (if diluted)	Time between sampling and pH measurement	pH
**Johnson et al. (1998)**	1 ml distilled water added to faecal sample (unspecified weight)	pH measured directly at sampling	6.0–6.8
**Nicol et al. (2002)**	4:1 (20 ml of distilled water: 5 g faeces)	Not given	6.05–6.58
**Hussein et al. (2004)**	none	pH measured on fresh mixed sample	6.64–7.04
**Rogers et al. (2004)**	1:1 (40 ml water: 40 g faeces)	pH measured within 2–8 h after faecal collection	5.83–6.72
**Zeyner et al. (2004)**	1:1 (35 g distilled water: 35 ml faeces)	Not given	6.36–6.68
**Berg et al. (2005)**	1:1 (double de**i**onized water)	pH measured directly at defecation	6.38–6.48
**Richards et al. (2006)**	1:1 (10 g distilled water: 10 ml faeces)	Not given	5.5–7.9
**Crawford et al. (2007)**	1:1 (2 ml deionized water: 2 g faeces)	Not given	6.18–6.97
**Williamson et al. (2007)**	1:1 (50 ml distilled water: 50 g faeces)	pH measured within 1 h of defecation	6.00–6.84
**Swyers et al. (2008)**	Not given	pH measured directly at sampling	6.50–6.63
**Muhonen et al. (2008)**	Pressed faecal liquid (no dilution)	pH measured directly on samples collected from rectum	6.4–6.6
**Müller et al. (2008)**	Pressed faecal liquid (no dilution)	pH measured directly on samples collected from rectum	6.07–6.36
**Müller (2009)**	Pressed faecal liquid (no dilution)	pH measured directly at defecation	6.39–6.87
**Willing et al. (2009)**	4:1 (20 ml water: 5 g faeces)	pH measured directly	6.63–6.79
**Goachet et al. (2010)**	Filtered through Blutex cloth 100 μm pore size (no information on dilution)	pH measured directly	6.5–7.1
**Næsset and Austbø (2010)**	1:1 (15 ml ion exchanged water: 15 g faeces)	pH measured directly on samples collected from rectum	6.26–6.80
**Müller (2012)**	Pressed faecal liquid (no dilution)	pH measured directly at defecation	6.27–6.83
**Siciliano and Schmitt (2012)**	1:1 (50 ml deionized water: 50 g faeces)	pH measured after 5 min equilibration of water: faeces mix	6.92–7.42
**van den Berg et al. (2013)**	300 g faeces	Faecal samples frozen at -20°C 1–2 h after sampling, thawed at room temperature (+20°C) before pH measurement	6.18–6.58
**Glunk et al. (2013)**	1:1 (50 ml deionized water: 50 g faeces)	pH measured after 5 min equilibration of water: faeces mix	7.21–7.67
**Jensen et al. (2013)**	1:1 (15 ml deionized water: 15 g faeces)	pH measured directly	5.85–6.21
**Hydock et al. (2014)**	No dilution (110–125 g faeces used for obtaining 30 ml faecal liquid) 1:1 (25 ml distilled water: 25 g faeces) 2:1 (20 ml distilled water: 10 g faeces) 3:1 (30 ml distilled water: 10 g faeces)	pH measured within 75 min of sampling. For all samples/dilutions the effect of equilibration time (0, 1, 3, 5 and 10 min) was evaluated	5.89–7.90
**Grimm et al. (2017)**	Filtered (no information on dilution)	pH measured directly at sampling	6.50–6.93
**Sadet-Bourgeteau et al. (2017)**	Filtered (no information on dilution)	pH measured directly at sampling	6.4–7.0
**Johnson and Rossow (2019)**	20 ml water added to faecal grab sample (unspecified weight)	pH measured within 10 min from sampling	6.89–7.03
**Lindroth et al. (2022) (sub-study I)**	Pressed faecal liquid (no dilution)	pH measured within 1 to 4 days, chilled samples + 4 to + 6°C	6.52–6.60
**Tischer et al. (2023)**	No dilution	pH measured on fresh samples and after: 24 h in + 4°C (refrigerator) 6 weeks in −20°C freezer 16 weeks in −20°C freezer Frozen samples thawed in 20–23°C water bath prior to pH-measurements	6.49 6.39 6.47 6.43
**Carter et al. (2025)**	No dilution, pressed faecal liquid through cheesecloth	Not given	5.58–6.70

Method descriptions and pH-values reported as given in the references.

## Materials and methods

According to the guidelines for studies that require ethical permits from ethical boards governing experiments on live animals in Sweden including the European Union Directives on Animal Experiments 2010/63/EU, Swedish Constitution 1988 534 and Swedish Board of Agriculture Constitution 2012 26, this study did not require any permit as faecal samples were collected after defecation, and the study had no influence on feeding, care or management of the horses.

### Faecal sample collection

Collection of fresh equine faecal samples was performed by the same operator (KML) from October 2018 to February 2019. Samples were collected on six different occasions from a total of 105 horses that were distributed over eleven horse farms in Uppsala County, Sweden. All horses were adult (mean ± SD age in years 12 ± 4.8) and of various breeds or types. The horses were kept in grass paddocks during the day and in individual boxes during the night, and they were fed forage-based diets (mean ± SD percent forage in total ration 96 ± 6.2). Samples (300 g) were taken from freshly deposited faeces on the stable floor from each horse within two minutes of defecation. To prevent contamination from the stable floor or bedding material, samples were taken from the centre of the faecal pile.

### Faecal sample treatment

At sample collection on farms, each faecal sample was placed in double plastic bags and mixed manually by kneading the sample from the outside of the bag before dividing it in 14 sub-samples of approximately 20 g each which were placed in new individual plastic bags. These sub-samples were subjected to different treatments where storage temperatures and time varied (Table 2). The pH-value was measured directly at sampling (in the stable) on one sub-sample which was considered to be the reference value (RT0). The remaining sub-samples were transported in a cooling bag with ice clamps (except RT2 which was not cooled) to the laboratory at the Department of Applied Animal Science and Welfare at Swedish University of Agricultural Sciences (SLU), Uppsala, Sweden, where they were stored in either a refrigerator (RE) at + 4 to + 6°C or a freezer (F) at -20°C according to the sample treatment protocol in Table 2. The time between sampling and placing samples in the refrigerator or freezer was one hour. Sub-sample RT2 was stored in room temperature during transport and at the laboratory before pH-measurement. Sub-samples stored in the refrigerator were measured for their pH-value at 2 (RE2), 4 (RE4), 8 (RE8), 12 (RE12), 24 (RE24) and 48 (RE48) h after sampling, and sub-samples stored in the freezer at 24 (F24), 48 (F48), and 72 (F72) h and 1 (F1M), 3 (F3M), and 6 (F6M) months after sampling (Table 2). Each sub-sample was only used for one treatment as all sample handling (removing from refrigerator or freezer and thawing) were considered to have a potential influence on pH (destructive sample handling and pH-measurement).

**Table 2:** Storage protocol and sample nomenclature for equine faecal sub-samples.

Storage time/temperature	0 h (At defecation)	2 h	4 h	8 h	12 h	24 h	48 h	72 h	1 month	3 months	6 months
**Room temperature (+20 to + 21°C)**	RT0 (reference sample)	RT2	–	–	–	–	–	–	–	–	–
**Refrigerator (+ 4 to + 6°C)**	–	RE2	RE4	RE8	RE12	RE24	RE48	–	–	–	–
**Freezer (−20°C)**	–	–	–	–	–	F24	F48	F72	F1M	F3M	F6M

### Measurements of pH

All pH-measurements were performed on liquid pressed from whole fresh, refrigerated or frozen faecal samples. Frozen samples were thawed in room temperature for 5 h prior to pressing. Samples were not diluted as it has previously been reported to increase variation in pH-value (Hydock et al. 2014). Faecal liquid was obtained by pressing the faecal sample in a hand-held stainless steel fruit press and collecting the liquid in a glass beaker, in which pH-measurement was performed using a portable pH-meter (WTW pH 315i, Wissenschaftliche Technische Werkstätten [WTW], Weilheim, Germany) with a glass electrode (WTW SenTix 41, WTW, Weilheim, Germany). The pH-meter was operated using the “Autoread” function to ensure signal stability before each reading. Before each measurement session, a two-point calibration of the pH-meter was performed according to the instructions by the manufacturer of the pH-meter by using buffer solutions with pH 4.00 and 7.00. The measuring range of the pH-meter was -2.00 to 16.00, the resolution was 0.01 pH units, and the accuracy was 0.01 pH units. In between pH-measurements of the samples, the electrode was rinsed with distilled water from a squeeze bottle. The pH-measurements were performed in a laboratory with a room temperature of + 20°C. The pH-value was read three times for each sub-sample, and the average of these readings, which were technical replicates, was used in the statistical evaluation of data.

### Statistical evaluation

Data were analysed using the software SAS 9.4 for Windows (SAS Institute, Cary, NC, USA) with an ANOVA mixed model procedure with sample treatment as fixed effect and horse as random effect. Residual plots showed similar variance and normally distributed data in all treatment groups. All treatments (Table 2) were compared to the reference value (RT0) in the statistical analysis using Dunnetts test for *P*-value adjustment to reduce Type I errors. Means were regarded as statistically different at *P* < 0.05. Means and SD within each sample treatment were calculated and reported together with lower and upper confidence levels and *P*-values.

## Results and discussion

Mean pH-values, SD, lower and upper confidence levels (CL) and *P*-values for comparisons of all sample treatments to RT0 are reported in Table 3. The mean pH-values ± SD ranged from 6.52 ± 0.36 to 6.75 ± 0.40 and were within the pH range reported in other studies of pH of equine faeces (Table 1). The lowest pH-value was 5.58 and the highest was 7.77, and these were within the range of faecal pH-values reported for horses in other studies summarized in Table 1. Faecal pH-value in horses differ with feed ration composition (Hussein et al. 2004; van den Berg et al. 2013), but also between (Zeyner et al. 2004) and within individuals (Jensen et al. 2013), and the pH-range in this study was considered as representative for horses fed forage-based diets.

**Table 3:** Mean, standard deviation (SD), and lower and upper confidence levels (CL) for pH-value in equine faecal samples stored in varying temperatures for different time periods, and *P-*value for contrast to reference value RT0 (pH measured directly at defecation).

Treatment	Mean	SD	Lower CL	Upper CL	*P-*value for contrast to RT0
**RT0**	6.60	0.34	6.53	6.67	reference
**RT2**	6.60	0.37	6.53	6.67	1.00
**RE2**	6.52	0.36	6.45	6.59	<0.001
**RE4**	6.52	0.36	6.45	6.60	<0.001
**RE8**	6.58	0.35	6.51	6.65	0.84
**RE12**	6.67	0.34	6.60	6.75	<0.001
**RE24**	6.67	0.38	6.59	6.74	<0.001
**RE48**	6.57	0.38	6.50	6.64	0.34
**F24**	6.69	0.37	6.62	6.77	<0.001
**F48**	6.65	0.38	6.58	6.72	<0.01
**F72**	6.75	0.40	6.67	6.82	<0.001
**F1M**	6.67	0.39	6.59	6.74	<0.001
**F3M**	6.68	0.38	6.61	6.75	<0.001
**F6M**	6.66	0.40	6.59	6.73	<0.001

Treatment abbreviations are explained in Table 1.

Treatments RT2, RE8 and RE48 were not different from RT0, while all other treatments differed from RT0 (Table 3). All F-treatments resulted in higher mean pH-values compared to RT0, while RE2 and RE4 samples had lower mean pH compared to RT0, and RE12 and RE24 samples had higher mean pH compared to RT0 (Table 3). A similar pattern of pH-value fluctuation in refrigerated and frozen samples was reported in soil samples in a study where sample storage conditions were identified as an important contributor to variation in pH-value, but no clear reason for it was discussed (Sollen-Norrlin and Rintoul-Hynes 2024). The reasons for the fluctuations in mean pH-value with varying storage time and temperature in the faecal samples in the current study may be several. Different biochemical pathways and enzyme systems in faecal microbes are inhibited by cooling and/or freezing, and salt precipitation may occur with freezing, which influences the pH in the solution (Hill and Buckley 1991). One factor that could explain the lower mean pH in RE2 and RE4 samples compared to RT0 may be a continued microbial activity during the first hours after sampling (Holter 1991; Lowman et al. 1999) before the temperature in the sample has decreased to the temperature in the refrigerator. This could result in an accumulation of SCFA, especially if the buffering system is restricted or postponed in its activity, which lowers the pH-value (Chang 1994). A gradually decreasing substrate availability in the sample may then cause microbes to start consuming SCFA and nitrogenous compounds of which the latter results in production of alkaline substances such as ammonia (Procházková et al. 2024). Both microbial consumption of SCFA and ammonia production results in a higher pH-value, which could explain why pH in RE8 samples increased again and were not different from RT0, and why RE12 and RE24 samples had higher pH-values compared to RT0 and RE8 samples. As temperature drops the pH-value may reach a steady state due to inhibition of microbial enzymes, but why the pH-value was lower again in RE48 (Table 3) is not clear. As pH is affected by for example the concentration of different SCFAs, and the p*K*_a_ of the SCFAs decreases with decreasing temperature (Olofsson 1984; Chang 1994), this may be a contributing explanatory factor to the lower pH in RE48 as a lower p*K*_a_ means a higher ionic strength in the solution (Chang 1994). Concentrations of SCFAs were not analysed in the faecal samples in the current study but could potentially explain some of the differences in mean pH-value between the different storage treatments. To fully understand why the mean pH-value first decreased and then increased in the refrigerated samples, concentrations of all pH-affecting substances in the samples would need to be analysed along with measuring pH, which was not possible in the current study.

The difference in mean pH between RT0 and RE- and F-treatments of the samples was from 0.05 to 0.15 pH-units in the dataset in this study. It may appear as a small and unimportant difference, but the difference in pH-units between horses with and without a hindgut disturbance such as acidosis has been reported to vary from 0.04 to 0.4 (Hussein et al. 2004; Berg et al. 2005; Williamson et al. 2007; Swyers et al. 2008) and there is thus a risk of not detecting this small difference with the large variation in sample handling prior to pH-measurement. Also, a difference of 0.1 pH-units corresponds to a 25% difference in the concentration of hydrogen ions (MacInnes et al. 1938; Westcott 1978), so even small changes in pH may be of relevance for the hindgut microbial community and it is therefore important that these small pH-changes are detectable, and that variation in pH-value that originates from sample handling is reduced as much as possible. In addition, it is also crucial to have a sufficient number of horses included in studies where faecal pH is the variable of interest. Hydock et al. (2014) showed that at least 25 horses were required in each group to be able to detect significant differences of 0.4 pH units between the groups, if the SD was not higher than 0.5, the statistical power 0.80 and α = .05. If the SD was 0.35, it would be sufficient with 15 horses in each group, illustrating the importance of reducing variation in the measurement of pH (Hydock et al. 2014). In the current study, the SD varied from 0.34 to 0.40 for the different sample treatments, and all treatments contained samples from 105 horses, meaning that the number of horses included was sufficient for detection of differences of at least 0.4 pH units.

Other sources of error are also present in pH-measurements in general, where the status of the electrodes in the pH-meter and the measuring technique have been reported to be the largest (Westcott 1978). Westcott (1978) reported errors of ±0.05 pH units that may arise from the pH-meter itself or the technique used at measuring. Wiping instead of blotting the electrode after rinsing it between sample readings can create a capacitance charge in the electrode which produces erroneous readings and may take up to 15 minutes to discharge. Another source of increased variation in pH-measurements in equine faeces is dilution of the samples with distilled water, compared to no dilution (Hydock et al. 2014). Dilution of faecal samples with water is commonly performed (Table 1) despite the reported increased variation in pH that follows from it (Hydock et al. 2014). Dilution was not performed in the current study due to the results presented by Hydock et al. (2014), but it could be of interest to investigate if faecal samples could be diluted with other fluids than distilled water prior to freezing to minimize pH changes associated with freezing of the samples (Hill and Buckley 1991).

In conclusion, the results of this study showed that it is of importance to provide information on sample handling prior to measurement of pH in equine faeces when reporting faecal pH-values, and to reduce variation in sample handling prior to pH-measurements. Measurement of pH in equine faeces should preferably be performed immediately at defecation on undiluted samples, but if that is not possible, storage of the sample in room temperature for 2 h or in a refrigerator at + 4 to + 6°C for 8 h or 48 h reduces the impact of sample storage on the pH-value in comparison with shorter or longer storage in a refrigerator. Storing equine faecal samples in a freezer from 24 h up to 6 months invariably resulted in higher mean pH-values compared to when measured immediately at defecation, and is therefore not recommended.

## Limitations of the study

The range of pH-values measured in this study was from 5.58 to 7.77, and the faecal samples were collected from clinically healthy horses fed forage-based diets. It is not known if the results of this study are valid for pH-values out of this range.

## List of abbreviations

pH: potential of hydrogen (H), *pondus hydrogenii*
p*K*_a,_: the negative log of the acid dissociation constant (Ka)
SCFA: short chain fatty acids
